# Orthorexia Nervosa in Bodybuilders: Exploring a Critical Research Gap and the Role of Social Media and Self-Monitoring Applications

**DOI:** 10.3390/ijerph23050640

**Published:** 2026-05-12

**Authors:** Francesca D’Apolito, Alessandra Bulbarelli, Elena Lonati, Paola Palestini, Emanuela Cazzaniga

**Affiliations:** 1 School of Medicine and Surgery, University of Milano-Bicocca, via Cadore 48, 20900 Monza, Italy; francescaa.dapolito@gmail.com (F.D.); alessandra.bulbarelli@unimib.it (A.B.); elena.lonati1@unimib.it (E.L.); emanuela.cazzaniga@unimib.it (E.C.); 2 Master’s Programme in Applied Food and Nutritional Sciences, School of Medicine and Surgery, University of Milano-Bicocca, 20900 Monza, Italy

**Keywords:** bodybuilding, orthorexia nervosa, social media, eating disorders, weight-related self-monitoring applications, body image, aesthetic sports

## Abstract

**Highlights:**

**Public health relevance—How does this work relate to a public health issue?**

- Bodybuilding athletes may represent a population vulnerable to disordered eating behaviors, including Orthorexia Nervosa (ON) due to extreme dietary restriction, body image centrality, and performance-driven aesthetic standards.
- Social media use and weight-related self-monitoring applications may contribute to the development or reinforcement of ON symptoms in vulnerable athletic populations.

**Public health significance—Why is this work of significance to public health?**

- ON symptomatology is associated with psychological distress, nutritional deficiencies, and comorbid eating disorders, potentially contributing to long-term mental and physical health burdens.
- Identifying bodybuilding as a potentially vulnerable population supports early detection strategies and broadens the understanding of eating disorder risk beyond traditionally studied groups.

**Public health implications—What are the key implications or messages for practitioners, policy makers and/or researchers in public health?**

- Early screening and education programs targeting athletes—particularly in aesthetic sports—can help prevent disordered eating behaviors and ON development.
- Future research should develop standardized diagnostic criteria and sport-specific assessment tools to improve surveillance, risk stratification, and public health policy planning.

**Abstract:**

Bodybuilding aims to achieve a muscular physique through intensive resistance training and strict dietary control. Athletes in this sport could be at risk of disordered eating behaviors; however, limited evidence exists regarding its association with ON. Social media use and Weight-Related Self-Monitoring (WRSM) applications may contribute to ON symptoms by reinforcing rigid dietary behaviors. These aspects remain underexplored in the current literature, particularly within the sporting context. This narrative review aimed to synthesize current evidence on the potential association between bodybuilding and ON, and to examine the influence of social media and weight-related self-monitoring (WRSM) applications on its development. Literature searches included the terms “bodybuilding AND orthorexia nervosa”, “bodybuilding AND eating disorders”, “bodybuilding AND social media”, “bodybuilding AND aesthetic pressure”, “orthorexia nervosa”, “orthorexia nervosa AND social media”, “fitness influencers” and “orthorexia nervosa in sport”. Available evidence suggests that bodybuilders may present risk factors for orthorexic tendencies, including dietary rigidity, body image centrality, perfectionism, and compulsive exercise. Social media may contribute by promoting unrealistic aesthetic standards, potentially reinforcing disordered eating patterns. Although direct data in bodybuilding are limited, findings indicate a plausible vulnerability profile. Monitoring and preventive strategies may be warranted to reduce the risk of onset or exacerbation of comorbid eating disorders.

## 1. Introduction

Competitive bodybuilding is a sport in which individuals display their physiques to a panel of judges, who score each entrant based on the size, symmetry and definition of the musculature [1]. To achieve this look, bodybuilders typically adopt rigid dietary rules, strict meal timing, cutting and bulking cycles and intensive supplement use [2,3]. While these practices are often normalized within bodybuilding culture, they may resemble maladaptive patterns characterized by excessive dietary control and perfectionism. Both competitive and non-competitive bodybuilders are preoccupied with their appearance and are particularly vulnerable to EDs (i.e., anorexia nervosa, binge eating disorder) as well as body image dissatisfaction, due to the strong emphasis on muscularity and low body fat levels [4,5]. Amateur bodybuilders can follow Do It Yourself (DIY) high-protein diets and consume excessive supplements (e.g., vitamins, minerals), which have been associated with gastrointestinal discomfort and other adverse health outcomes. In preparation for competitions, athletes may also adopt methods that are potentially dangerous to health (e.g., rapid weight-loss strategies or extreme dietary restriction) [6,7]. In this context, the emphasis on “clean eating”, discipline and absolute nutritional control may evolve into dysfunctional eating behaviors, particularly when healthy eating shifts into compulsive food selection and avoidance behaviors.

ON is an excessive preoccupation with consuming only biologically pure foods and eating in a healthy way, leading to dietary limitation, malnutrition, and underweight [8]. This rigid dietary behavior is often associated with a substantial impact on daily functioning and social life, as individuals devote considerable time and cognitive effort to planning, purchasing, and preparing meals, while experiencing guilt and self-punishment when they fail to adhere to their self-imposed dietary rules [9]. In addition, in orthorexic individuals, the accomplishment of the self-imposed eating behavior excessively determines the body image, self-worth, identity and/or satisfaction [9].

ON is not currently recognized as a formal psychiatric disorder in the Diagnostic and Statistical Manual of Mental Disorders or the International Classification of Diseases [10,11,12], but studies suggest a reciprocal association between ON and ED-related symptomatology, and even negative image and excessive exercise may exacerbate ON symptoms [13,14]. In the assessment of ON, the ORTO-15 questionnaire is commonly used as a screening tool for ON; however, the lack of standardized cut-off values (ranging from 35 to 45 points across studies) may lead to inconsistent classification and potential misdiagnosis [9,13]. The Düsseldorf Orthorexia Scale (DOS) has been proposed as an alternative with improved psychometric properties, although no consensus exists regarding gold-standard measurement [15].

The global prevalence of ON is approximately 30%, with no clear differences between men and women [13]. Although this disturbed eating pattern is widely studied globally, little is known about its prevalence in sports, including bodybuilding, where excessive exercise is common in order to achieve optimal physique for competition. First, regarding athletes, existing evidence suggests that ON tendencies may be more prevalent in sports characterized by weight or aesthetic demands. Studies conducted in weight-dependent and aesthetic disciplines (e.g., figure skating, dance) report prevalence rates ranging from 41.3% to 88.3%, depending on the assessment criteria used [16,17]. In addition to that, orthorexic traits have been demonstrated in people who practice fitness sports and users of social media [18]. These findings indicate that environments emphasizing body control, dietary restriction, and performance optimization may represent a relevant risk context for orthorexic behaviors. Second, regarding social media, a growing body of research indicates that digital platforms play a significant role in shaping eating behaviors and body image. Social media can promote health-related information but also reinforce unattainable ideals, dietary rigidity, and “clean eating” trends [19,20]. For bodybuilders, social media may be especially influential because it continuously reinforces appearance-based standards through physique comparisons, fitness influencers and nutrition-related contents [21,22,23]. Furthermore, a significant relationship between symptoms of ON and Instagram use has been reported, with higher engagement on this image-focused platform associated with a greater tendency towards ON [24,25]. Weight-Related Self-Monitoring (WRSM) smartphone applications may further reinforce rigid dietary control by promoting continuous tracking of calories, macronutrients and physical activity targets [26]. In bodybuilding populations, these tools may intensify obsessive monitoring behaviors and perfectionistic tendencies associated with ON. However, despite these advances, important gaps remain. Specifically, evidence on ON in bodybuilding populations is still scarce and underdeveloped. Bodybuilding represents a unique context in which strict dietary control, physique monitoring, and performance-related body modification strategies are not only common but culturally reinforced. Unlike other aesthetic sports, bodybuilding places an extreme and sustained emphasis on muscularity, leanness, and visual evaluation, which may uniquely interact with orthorexic tendencies.

Despite these potential risks, the combined role of bodybuilding, social media, and self-monitoring technologies in the development of ON remains insufficiently explored. Specifically, although evidence exists on orthorexic tendencies in sports and on the relationship between social media and disordered eating in general populations, these findings have not yet been synthesized with a specific focus on bodybuilding populations. Given the increasing popularity of bodybuilding among young adults, this topic is relevant for prevention and early identification strategies. Therefore, this narrative review aims to address this gap by synthesizing the limited available evidence on orthorexia nervosa in bodybuilding populations and by exploring the potential role of social media and WRSM applications as contextual factors. Considering the limited literature specifically focusing on bodybuilding, evidence from other aesthetic and appearance-focused sports will be considered to provide useful insights and inform future research directions in this field.

## 2. Materials and Methods

**Figure 1:**
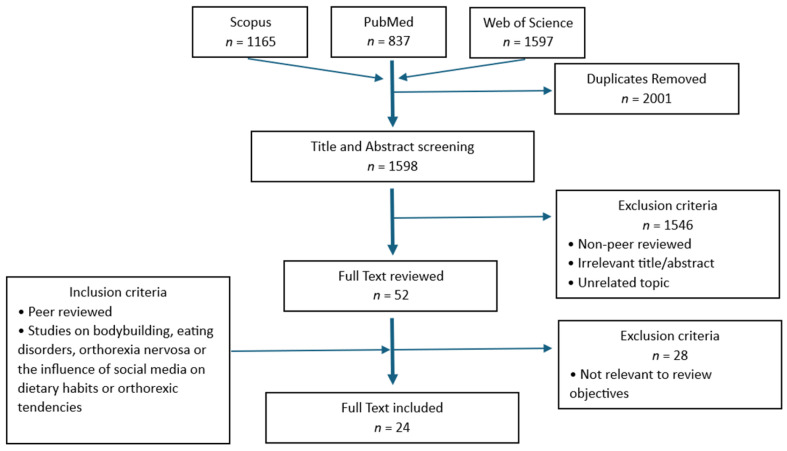
Flow diagram describing the search methodology used to obtain the research studies for this review.

This study is a narrative review. The literature search was conducted from January to April 2026. This approach was chosen due to the scarcity of literature regarding the potential correlation between bodybuilding and ON, as well as the role of social media and WRSM applications within this context. A narrative review facilitates a comprehensive synthesis of current scientific knowledge while allowing for the integration of findings on related sports, where body weight and aesthetic form play a key role, given the lack of studies specifically analysing the bodybuilding context. PubMed was used as the primary database due to its focus on biomedical literature, while Scopus and Web of Science were consulted to capture a broader range of interdisciplinary publications and to reduce the risk of database selection bias. Search terms were combined using Boolean operators (AND/OR) to refine results, focusing on studies addressing the intersection between bodybuilding and orthorexia nervosa, or related influences of social media on eating behaviors in aesthetic sports. The search terms used for the analysis were ‘bodybuilding AND orthorexia nervosa’, ‘bodybuilding AND eating disorders’, ‘bodybuilding AND social media’, ‘bodybuilding AND aesthetic pressure, ‘orthorexia nervosa’, ‘orthorexia nervosa AND social media’, ‘fitness influencers’ and ‘orthorexia nervosa in sport’. (Figure 1). The results of the searches conducted in each database are presented in Table 1. Study selection followed a structured multi-step process: titles were screened first, followed by abstract review, and then full-text assessment for relevance. No formal quality assessment tool was applied due to the narrative nature of the review. The inclusion criteria were limited to peer-reviewed articles published in English that examined bodybuilding, eating disorders, ON, or the influence of social media on dietary habits or orthorexic tendencies. Review articles were included to support the theoretical background and contextualize the findings from empirical studies. Non-peer-reviewed materials and studies were excluded, as well as those with irrelevant titles or abstracts, redundant or repetitive findings, unrelated topics, and duplicates. Generative artificial intelligence (GenAI) tools were used as a supplementary support to assist in the identification and cross-checking of duplicate records. The literature considered in this review spans from June 2005 to April 2025. This temporal range was chosen to capture the emergence and development of orthorexia nervosa as a construct in scientific literature, as well as the subsequent growth of research on social media and aesthetic sports. A total of 24 peer-reviewed articles met the inclusion criteria and were included in the final analysis. In addition, 3 books were retained as supplementary background sources and are not included in the analytical synthesis; therefore, Table 2 reports a total of 24 references overall. Table 2 summarizes the studies analysed for this review.

## 3. Bodybuilding: A Sport at Risk for Eating Disorders

Bodybuilding is the pursuit of a muscular physique through a regime of weight training and a tailored programme of nutrition [1]. In order to achieve high levels of muscularity and symmetry combined with low levels of body fat, bodybuilders use strategies that involve rigorous exercise practices, as well as dietary manipulations including energy restriction and supplementation, depending on the phase of the athlete’s competitive cycle [2]. This cycle consists of two primary phases, termed “bulking” and “cutting”. During the bulking phase, which takes place at the beginning of contest preparation, the athlete’s diet usually contains more calories, mainly derived from carbohydrates and proteins. On the other hand, the cutting phase is initiated during the last months before the contest and is characterized by energy-deficient and nutritionally imbalanced diets [2]. In this context, bodybuilders typically train for years to increase muscle mass, then enter a diet phase, with the aim of reducing body fat to extremely low levels while maintaining high levels of muscularity [3].

Competitive bodybuilding is not only about the performance, but rather literally about appearance. In fact, competitive and non-competitive bodybuilders have a preoccupation with how they appear and are particularly vulnerable to eating disorders and body image dissatisfaction as a consequence of the obsession with being more muscular and leaner [4].

Common EDs among bodybuilders are anorexia nervosa (AN) and binge eating disorder (BED). In addition, professional and recreational bodybuilders of both sexes have higher levels of neuroticism, perfectionism, obsession and need for control, but also depression and anxiety, in comparison with strength athletes, who do not perform bodybuilding [5]. Studies have linked the overwhelming passion, in both competitive and recreational bodybuilders, for a lean, heavily muscled body, with the risk of EDs and pathological symptoms. This risk was associated with the desire for a lower body weight, in both professional and recreational athletes. It is important to point out that recreational athletes are more vulnerable to disordered eating behaviors, because professional athletes have the help of coaches who are well-informed and sensitized regarding the complications of EDs [5]. On the other hand, amateur bodybuilders can follow DIY diets and consume supplements exceeding by far the recommended daily intake levels. This leads to an overconsumption of some macro and/or micronutrients, exposing athletes to potential health risks [6]. Despite this, even professional bodybuilders are at risk, because those preparing for a contest can use methods dangerous to health. These methods include chronic energy restriction, dehydration (water manipulation), sporadic eating and inappropriate use of diuretics, anabolic steroids and ‘fat burners’. This can lead to physiological (i.e., decreased bone mineral density, metabolic disruption, increased cardiovascular strain), hormonal and/or psychological consequences, among which loss of eating control/binge eating and preoccupation with food [7]. These findings suggest that within bodybuilding, behaviors typically considered maladaptive in other contexts may become normalized or even reinforced, potentially obscuring early signs of disordered eating and delaying recognition of clinically relevant conditions.

Bodybuilding presents myths and practices that are contrary to scientific literature, which can lead to various health problems. Adopting a scientifically designed approach is very important, as it can help bodybuilders achieve better results while preserving their health. At the same time, preventing the risk of a possible eating disorder is of paramount importance for these athletes.

## 4. Orthorexia Nervosa: Diagnostic Challenges, Prevalence and Risk Factors

The term orthorexia nervosa was coined by Bratman to describe an excessive preoccupation with consuming only biologically pure foods and eating in a healthy way, leading to dietary limitation, malnutrition, and underweight [8,9]. Since its introduction, ON has been described as a condition characterized by rigid and extreme dietary rules, although its conceptual boundaries remain debated. People with this problem avoid having foods considered “impure”, unhealthy or contaminated and this preoccupation, when extreme, leads to restricted social lives. They spend excessive amounts of money and time buying food and planning, preparing and consuming meals, and if they do not follow their rigid self-imposed rules, they feel guilty and punish themselves [9].

Previous studies found significant associations between ON scores and both eating disorder symptomatology and obsessive compulsive traits and symptoms [9]. These findings suggest that ON shares features with both EDs and obsessive compulsive symptomatology, complicating its classification as a distinct condition. However, ON is not recognized as a disorder by the Diagnostic and Statistical Manual of Mental Disorders [10] or by the International Classification of Diseases [11].

Several instruments have been developed to evaluate ON, although important methodological limitations remain. The ORTO-15 test is considered the gold standard in the assessment of ON, but concerns have been raised regarding its diagnostic accuracy, particularly due to the lack of standardized cut-off values, which range from 35 to 45 across studies [9,12,13]. Other instruments, such as the Bratman test and the Eating Habits Questionnaire (EHQ), have also been criticized for limited validation or for not adequately capturing the emotional distress associated with ON [9]. The Düsseldorf Orthorexia Scale (DOS) has been proposed as a more robust alternative, with defined cut-offs for identifying both the presence of ON and individuals at risk [15]. The heterogeneity of assessment tools reflects the absence of a shared definition of ON and raises concerns about its diagnostic reliability. This lack of consensus in measurement further complicates the interpretation of prevalence rates and associated risk factors reported in the literature.

As already stated, ON is characterized by a strong need to eat pure and healthy foods, with the motivation to attain a feeling of perfection or purity, but this is further complicated by the fact that they perceive this obsession as normal and even healthy [13]. Proposed diagnostic criteria for ON include: (i) persistent concerns about healthy eating or food quality; (ii) the adoption of rigid food rules and heightened emotional distress following perceived dietary transgressions; and (iii) impairment of daily life activities resultant from obsessive dietary patterns [14]. However, these criteria have not been universally adopted, further highlighting the ongoing debate regarding the clinical status of ON. Body image plays a key role in the development of ON, as it often forms the basis for the creation of unhealthy eating patterns. In addition, both negative image and excessive exercise can reinforce each other, leading to the exacerbation of ON symptoms. Bodybuilders may over-exercise, and this often leads to obsessive focus on body image, which can deepen the ON-related complications [13]. These findings suggest a self-perpetuating cycle in which body image concerns and excessive training reinforce one another, increasing vulnerability to rigid and potentially maladaptive eating and exercise behaviors.

It is important to note that higher ON symptoms are associated with increased eating concerns and more frequent binge eating and driven exercise episodes but also increased depressive symptoms over time. Consequently, this disturbed eating pattern may lead to some worsening of ED-specific and general mental health symptoms. Furthermore, eating disorders such as anorexia nervosa and bulimia nervosa significantly increase the risk of developing orthorexia [13,14]. The literature suggests that orthorexic tendencies may emerge from a complex interplay between body image concerns, rigid behavioral control, and eating disorder-related psychopathology, rather than from isolated risk factors. These characteristics may be particularly relevant in sport contexts, where dietary control and body composition are closely linked to performance and aesthetics. While the global prevalence of ON symptoms is about 30%—affecting men and women equally—little is known about its prevalence in sport [13,14,15,16]. A 2020 study evaluating a cohort of Olympic athletes, including weight dependent sports (figure skating, judo, rowing lightweight, taekwondo), found that, depending on the adopted cut-off value of 35 or 40 points, 41.3% to 88.3% of participants, respectively, manifested ON tendencies. In this subgroup, factors related to ON tendencies concerned Body Mass Index (BMI) and Bone Mineral Content (BMC), though only in male athletes [16]. Similarly, 74% of Greek professional dancers exhibited symptoms of ON, correlated with BMI and body image inflexibility [17]. In this sport, physical fitness and body aesthetics play an important role, just like in bodybuilding, although artistic performance is also a key component for dancers. Moreover, orthorexic behavior has been demonstrated in individuals strongly focused on a healthy lifestyle, such as yoga practitioners, fitness sports and users of social media; nonetheless, in sports settings, this relationship is still not summarized [18]. This suggests that it may not be limited to competitive sport, but also extended to digitally mediated environments where health and fitness ideals are continuously reinforced. In the case of athletic performance in aesthetic sports, lower body weight has a beneficial effect, but to achieve this goal athletes go on diets resulting in low energy availability, negatively affecting physiological functions needed for optimal health. This behavior is linked to the concept of Relative Energy Deficiency in Sport (RED-S) that IOC (International Olympic Committee) introduced recently and addressed serious health issues in both male and female athletes [18]. Bodybuilding, given its nature focused on physical image and extreme levels of exercise, should be monitored for the risk of developing ON or orthorexic behaviors, which may contribute to the onset or worsening of comorbid eating disorders. Overall, these findings suggest that orthorexic tendencies may be especially prevalent in performance- and appearance-oriented contexts, although heterogeneity in assessment methods limits the comparability of results across studies and may contribute to inconsistent prevalence estimates.

## 5. Orthorexia in the Age of Social Media

The internet has become the largest and fastest-growing source of health information, with millions of individuals conducting daily searches. Social media platforms wield significant social influence, empowering users to express opinions on critical issues and shaping attitudes and perceptions on a broad range of topics. Platforms have emerged as powerful tools for sharing information, discussing healthcare practices and promoting health behaviors [19]. Within this context, social media also play a central role in shaping food- and body-related attitudes through the continuous exposure to appearance- and health-related content. The widespread use of social media has amplified discussion around food and diet, leading to a significant rise in nutrition-related searches. The interactive nature has exacerbated the spread of misinformation—false information shared by individuals who believe it to be true—and disinformation—intentionally false information shared to mislead others [20].

Health and fitness accounts have become relevant in people’s lives, often followed due to personal interest in health and fitness. Once social media users participate in a community interested in health and fitness content, their interest in personal health is likely to increase, resulting in increased engagement in health- and fitness-related behaviors such as controlling which food types to eat [20]. Followers of health and fitness accounts were found to have higher thin-ideal internalization and drive for thinness, and this exposure negatively influences young adults’ body image and food choices. In addition, users’ involvement with health and fitness accounts on social media is positively related to ON tendencies, and also with thin-ideal and muscular internalizations [20]. These dynamics suggest that social media are characterized by environments in which health-oriented content progressively shifts toward rigid and appearance-focused dietary and exercise behaviors. The evidence suggests that individuals’ movement to ON tendencies has been associated with time spent on healthy eating blogs and social media sites [21]. These platforms are full of reflections and discourses that may contribute to individuals’ understanding of ‘good’ and ‘bad’ food or lean bodies and ‘best’ identities. It has been noticed that some sites potentially normalize disordered eating behaviors and encourage orthorexia-type behaviors, sharing lists of ‘impure’ or ‘safe’ foods and an extreme fear and worry of ‘food impurity’ that could potentially encourage users to practice self-starvation. Furthermore, these sites can show users dieting ‘tips and tricks’ or photo/video galleries providing ‘thininspiration’ for extreme weight loss, suggesting calorie restriction and excessive exercise, while signals of hunger are diminished in value [21]. At the same time, authors of these websites can sell products, using adverts and affiliated links for books with low-carbs recipes, home bikes, supplements and weight loss pills [21]. This is consistent with the idea that they operate within a commercialized digital ecosystem where lifestyle content, body ideals, and dietary practices are strategically optimized for engagement and monetization rather than evidence-based health communication. This may increase their exposure to online content that promotes rigid dietary rules, extreme nutritional control, and idealized body standards, which are frequently disseminated through fitness and body transformation communities.

The processes involved in this association appear to be different and may include cognitive and psychological processes (e.g., internalization of appearance ideals, social comparison, dichotomous thinking), behavioral factors (e.g., normalization of extreme behavior, behavioral reinforcement), but also socio-economic influences (e.g., sponsored content, product endorsement). These processes may interact within a broader digital environment shaped by algorithmic curation, user engagement, and commercial incentives, which may be associated with increased exposure to appearance-focused content.

Fitness influencers share workout routines, body image ideals, lifestyle representation, and dietary practices. Their content is often in the form of short videos featuring “before-and-after” transformations, showing that willpower, training consistency and bodily transformation are the formula for success [22]. In addition to these content-related processes, platform-specific features may further amplify exposure. For instance, Instagram and TikTok differ in both content structure and user engagement patterns. Instagram, image-based and characterized by curated visual feeds, tends to promote static idealized representations, whereas TikTok, based on short-form video [23], facilitates rapid and repetitive exposure to appearance- and fitness-related material.

Through their content, fitness influencers help construct perceived norms of bodily aesthetics, promoting specific expectations regarding appearance. Females are more sensitive to media-propagated ideals of thinness, whereas males are more likely to internalize muscularity ideals and aspire to a “sculpted” physique. In both cases, fitness content creators can spread pressures that reinforce unrealistic standards, consolidating the false belief that physical perfection is a universal ideal that must be achieved. It is important to point out that these content creators typically do not hold relevant health or fitness qualifications [22,23]; this makes it even more important to conduct research into this entirely contemporary aspect of their influence, particularly regarding the impact that social networks and influencer culture may have on users’ health.

Among the various trends on social media, fitspiration has gained significant traction. It encompasses images and video that aim to inspire individuals to live an active and healthy lifestyle through diet and exercise, but the fit ideal is unrealistic and unattainable for most people [23]. This type of content often presents idealized physiques and strict dietary and exercise norms. At a conceptual level, such representations may be interpreted as promoting perfectionistic standards and may be related to more rigid approaches to diet and exercise aimed at achieving idealized body images. Users may report engaging in highly demanding efforts to achieve physical ideals, although these efforts do not always result in the desired outcomes, sometimes being accompanied by increasingly strict dietary practices.

Differences between platforms may further explain variations in user exposure and behavioral impact. For example, Instagram contains a high volume of fitspiration content and has a global user base, whereas TikTok is characterized by higher daily usage time and highly personalized algorithmic feeds [23]. Both platforms allow interaction through likes, comments, and sharing; however, TikTok’s algorithm facilitates the discovery of new hashtags and content categories, increasing exposure to fitspiration-related material [23].

The use of hashtags further facilitates the categorization and dissemination of disordered eating-related content, enabling the circulation of different types of content, including material related to extreme or maladaptive eating practices.

This exposure may be potentially harmful due to the presence of body shaming, excessive dieting, and the glorification of disordered eating behaviors. An additional relevant aspect to consider is that substantial proportion of fitspiration content depicts individuals with average body sizes but highly defined muscular physiques [23]. As active users of social media, bodybuilders are therefore directly exposed to content that reinforces unrealistic body ideals and may be associated with higher levels of disordered eating and orthorexic tendencies.

A study by Turner et al. (2017) [24] reveals a significant relationship between symptoms of ON and Instagram use, with higher use of this platform associated with a greater tendency towards ON. Instagram has an image-focused nature where eating ‘celebrities’ can influence large numbers of individuals by posting images portraying a certain diet or behaviors. An exploratory analysis into other social media channels showed that also Twitter had a positive association with ON symptoms [24].

Social media have a dual role: they can aid recovery as well as stimulate pro-EDs thoughts. Positive effects include facilitating the acquisition of information about recovery and reducing stigma. These are counterbalanced by negative effects, like triggering EDs symptoms or promoting comparison [25].

Being the platform of choice of the healthy eating community, Instagram has been the social media platform most studied with regard to ON. After Instagrammer Jordan Younger confessed to suffer from ON, this concept began spreading among the general population online. After that, Santarossa et al. analysed the hashtag #*orthorexia* on Instagram, and identified a relatively small but supportive community encouraging recovery [25]. Individuals seem to emulate each other through interaction on Instagram, but unfortunately, this study reveals a negative contagion, which may contribute to the spread of EDs [25]. In addition, hashtags can be understood as a process that facilitates the dissemination of eating disorder-related content, as they enable rapid aggregation and access to large volumes of topic-specific posts.

Although still less studied, the use of WRSM smartphone applications, such as FitBit and MyFitnessPal, can lead to the development of eating disorders. In fact, there is a strong association between WRSM app use and disordered eating behaviors [26]. These applications may promote excessive self-monitoring and rigid goal-oriented eating patterns, reinforcing cognitive preoccupation with food intake, body weight, and performance outcomes. Within this framework, self-tracking may shift from a health-promoting behavior to a compulsive form of self-regulation, increasing vulnerability to rigid dietary patterns and orthorexic tendencies. Some common weight-control behaviors are fasting and purging, while muscle-building behaviors are the use of steroids, protein powders and other muscle-building substances. This can be encouraged by the respective app types, for example, dietary-focused apps emphasize meeting nutrient goals, so individuals may use substances (e.g., protein powders) to meet dietary targets [26]. In this regard, bodybuilders frequently take protein supplements, but also amino acids, vitamins and minerals [27]. With the exception of protein supplements, these do not provide energy in terms of calories, but nutrients useful for various biological processes that can still be included in such applications. Importantly, WRSM applications should be conceptualized as part of a broader digital ecosystem of health and fitness tracking, where quantification, optimization, and constant feedback may reinforce appearance- and performance-driven behaviors through mechanisms of self-monitoring and behavioral reinforcement.

The pervasive influence that social media have on their users is clear. The risk of developing disordered eating behaviors is significant, particularly regarding ON symptoms. Athletes, and bodybuilders in particular, represent a high-risk group as they exhibit thin-ideal and muscular internalizations, which can be further exacerbated by the constant digital reinforcement of these ideals. Social media should be conceptualized not merely as a passive source of information, but as an active socio-digital environment shaping cognitive, behavioral, and cultural mechanisms underlying disordered eating risk in appearance- and performance-focused populations.

## 6. Discussion

Given the absence of studies directly investigating orthorexia nervosa in bodybuilding populations, the present discussion adopts an indirect evidence approach, integrating findings from aesthetic and weight-sensitive sports to propose a theoretically informed interpretation of potential risk patterns in bodybuilding. This is a sport in which physical appearance is crucial. In fact, bodybuilding culture is characterized by the desire to become leaner and more muscular at the same time. This may plausibly contribute to increased vulnerability to EDs and body image dissatisfaction. This aesthetic pressure may create a context in which restrictive and compensatory eating behaviors are not only frequent but may also become socially normalized within the sport. The role of coaching appears ambivalent, functioning both as a potential protective factor and as a source of reinforcement of extreme dietary and training practices, depending on the level of awareness and evidence-based knowledge.

While several studies have investigated bodybuilding in relation to EDs such as AN and BED, limited information is available regarding ON, highlighting a gap in understanding rigid dietary control in this context. To date, the literature specifically addressing orthorexia nervosa in bodybuilding populations remains scarce and fragmented. This prevents drawing definitive conclusions about the prevalence and characteristics of ON in this specific group. Although not specific to bodybuilding, these patterns are highly consistent with the rigid dietary control typically observed in bodybuilding contexts.

ON is not classified as an ED and the diagnostic criteria are still discussed. The gold standard for the assessment of orthorexic tendencies is the ORTO-15 questionnaire, but there is no standardization for the cut-off. This lack of consensus leads to substantial variability in prevalence estimates and limits the comparability of findings across studies. The Düsseldorf Orthorexia Scale (DOS) represents another widely used instrument. Compared with ORTO-15, the DOS is a shorter and more recently developed tool, designed to capture orthorexic behaviors and attitudes through a unidimensional structure. However, it does not fully resolve the conceptual and diagnostic ambiguity surrounding orthorexia nervosa. The coexistence of different assessment instruments and variable cut-off scores further supports the notion that orthorexia nervosa is currently operationalized through partially overlapping tools rather than a unified diagnostic framework. This limitation is particularly relevant when attempting to interpret orthorexic tendencies in structured dietary contexts such as bodybuilding, where dietary restrictions may overlap with sport-specific practices.

Attention should be paid to this restricted eating pattern, as it may lead to some worsening of ED-specific and general mental health symptoms. At the same time, the presence of EDs significantly increases the risk of developing ON. Evidence specifically addressing ON in bodybuilding populations remains limited, but there is some evidence of a high prevalence of ON in sports where physical fitness and body aesthetics play an important role. The available evidence suggests that orthorexic tendencies may be particularly relevant in appearance-focused sports. However, the lack of bodybuilding-specific investigations highlights the need for more targeted research in these settings.

The studies included in this review also suggest a relevant role of digital environments. Food- and diet-related contents are widely disseminated across social media platforms, which function not only as information sources but also as interactive environments that actively shape health-related perceptions and behaviors. Rather than operating in a neutral informational context, these platforms structure exposure through algorithmic curation, engagement-based amplification, and socially driven content diffusion. In this framework, health and fitness accounts are generally associated with adverse body image and eating-related outcomes in their followers and are positively related to ON tendencies. Instagram use has also been related to ON, with higher use associated with greater symptoms levels. Furthermore, the use of WRSM smartphone applications has been linked to disordered eating behaviors, but this area is still less investigated. From a theoretical perspective, these applications may promote continuous self-monitoring and quantification practices, which are hypothesised to be linked to increased cognitive preoccupation with food intake and body-related metrics in contexts characterised by strict dietary control. The influence of social media may be better understood as a complex system in which cognitive, behavioral, and platform-level factors interact to normalize and reinforce disordered eating-related patterns. In bodybuilding contexts, where physique monitoring and dietary precision are already central, these digital dynamics may not only reinforce but also legitimize extreme behaviors as normative practice.

From a practical perspective, these findings suggest the need for targeted preventive strategies within bodybuilding environments. Coaches may play a key role by promoting flexible, evidence-based nutritional approaches and by avoiding the reinforcement of excessively rigid dietary rules. Athletes may benefit from increased education on the distinction between performance-oriented dietary practices and maladaptive patterns characterized by excessive rigidity and preoccupation with food quality. Health professionals should be aware of the potential overlap between sport-specific dietary control and early orthorexic tendencies, in order to improve early identification and provide appropriate guidance. Additionally, digital literacy interventions may help individuals critically evaluate social media content related to diet and fitness, potentially reducing the internalization of unrealistic or extreme norms.

## 7. Limits of the Study

This narrative review presents several limitations that should be acknowledged. First, the literature specifically addressing ON in bodybuilding is extremely limited, as most findings are extrapolated from broader studies on EDs, aesthetic sports, or weight-dependent disciplines. These populations may not fully capture the unique psychological, cultural, and physiological characteristics of bodybuilding, limiting the specificity and interpretability of the evidence in this population. Second, many of the studies reviewed rely on self-report questionnaires, particularly the ORTO-15. However, its psychometric limitations and inconsistent cut-off scores reduce diagnostic reliability. This may lead to both overestimation or underestimation of ON prevalence, introducing substantial measurement bias across studies. Finally, heterogeneity in study populations (e.g., professional vs. amateur athletes, male vs. female participants) and variability in definitions of disordered eating behaviors limit comparability across studies and hinder the possibility of drawing consistent conclusions across the existing literature. These methodological constraints highlight the need for more robust and standardized research approaches to improve comparability, validity, and generalizability in future studies on orthorexia nervosa in bodybuilding population.

## 8. Conclusions

Bodybuilding may represent a theoretically relevant context for the development of orthorexia nervosa due to its emphasis on extreme leanness, dietary precision, and body image centrality. However, direct evidence on orthorexia nervosa in bodybuilding populations is currently lacking. For this reason, the present interpretation is necessarily indirect and based on findings from related literature on aesthetic and weight-dependent sports, where orthorexic tendencies have been more frequently investigated. Rather than indicating a confirmed risk, the available evidence suggests that bodybuilding shares structural characteristics with these sports, including aesthetic pressure, performance-oriented dietary control, and increasing exposure to digital fitness environments. These overlapping features may justify further investigation into orthorexic tendencies within this population.

The expanding influence of social media and weight-related self-monitoring applications may further exacerbate rigid eating patterns and reinforce maladaptive perfectionistic tendencies; however, their specific impact in bodybuilding populations remains underexplored and should be interpreted with caution. Rather than providing direct prevalence estimates, this review contributes by identifying a convergence of risk factors across adjacent domains, suggesting that bodybuilding may represent a potentially high-risk but currently understudied context for orthorexic tendencies. In conclusion, orthorexic tendencies in bodybuilding should be considered a hypothesized risk domain rather than an established phenomenon, warranting targeted empirical investigation.

## 9. Future Directions

Future research should prioritize the development and validation of standardized, sport-sensitive diagnostic criteria for orthorexia nervosa, particularly tailored to bodybuilding populations. Distinguishing between performance-oriented dietary discipline and clinically significant pathological rigidity is essential to avoid both under- and over-pathologization. In this context, future work should also directly compare existing assessment tools (e.g., ORTO-15, DOS and other emerging instruments) in order to clarify their convergent validity and applicability in sport-specific populations.

Longitudinal studies are needed to clarify temporal and causal relationships between bodybuilding practices, social media engagement, weight-related self-monitoring application use, and the onset or progression of ON symptoms.

Further investigations should also explore gender differences, competitive level (amateur vs. professional), and cultural influences, as well as the potential moderating role of coaching supervision and nutritional guidance. In addition, future research should examine the role of sport-specific contextual factors in bodybuilding (e.g., contest preparation phases, off-season vs. pre-competition periods), which may differentially influence orthorexic tendencies.

Finally, future studies should consider integrating multidisciplinary frameworks combining sports science, clinical psychology, and digital behavior analysis, in order to better capture the complex and multifactorial nature of orthorexic tendencies in contemporary sport environments.

## Figures and Tables

**Table 1:** Number of records retrieved from each database search.

Search String	PubMed	Web of Science	Scopus
bodybuilding AND orthorexia nervosa	3	106	3
bodybuilding AND eating disorders	55	79	55
bodybuilding AND social media	25	50	45
bodybuilding AND aesthetic pressure	1	37	1
orthorexia nervosa	569	775	733
orthorexia nervosa AND social media	50	150	83
fitness influencers	66	255	190
orthorexia nervosa in sport	68	145	55
Total	837	1597	1165

**Table 2:** Summary of the cited studies.

References	Population (No.)	Research Design	Aim of the Study	Outcomes
[1]	no empirical sample	narrative review	to review the concept of muscle dysmorphia in bodybuilding context	muscle dysmorphia involves distorted body image, compulsive training behaviors, and maladaptive beliefs about muscularity
[2]	Men’s Physique competitors (16)	prospective observational study	to compare the dietary routines of bodybuilders during “bulking” and “cutting” phases	decrease in the intake of most macro- and micro-nutrients from bulking to cutting
[3]	bodybuilding coaches (33)	cross-sectional qualitative study	to understand the common decisions and rationale employed by bodybuilding coaches	bodybuilding coaches’ decisions partially align with current evidence-based recommendations
[4]	bodybuilders (62 competitive, 58 noncompetitive)	cross-sectional observational study	to determine the relationship between body image disturbance and eating disorders in male bodybuilders	Eating Attitudes Test (EAT) is positively correlated with fat and muscle dissatisfaction
[5]	bodybuilders (professional 38, recreational 32)	cross-sectional study	to investigate potential exacerbators on the development of disordered eating in bodybuilding and strength athletes	recreational bodybuilders are more vulnerable to disordered eating behaviors
[6]	amateur bodybuilder (1)	case report study	to document the development of possible adverse effects in a amateur bodybuilder who consumed for 16 years a DIY high-protein diet associated with nutrient supplementation	presence of permanent abdominal discomfort
[7]	amateur bodybuilder (1)	case report study	to describe the effects of a structured nutrition and conditioning intervention on body composition, performance and physiological responses in a single amateur bodybuilder during contest preparation	a structured and scientifically supported nutrition strategy can help to improve parameters relevant to bodybuilding competition and the health of competitors
[9]	university students (sample 1: 807; sample 2: 242)	psychometric validation study	to analyze the psychometric properties of the Spanish adaptation of the ORTO-15	the psychometric properties of the Spanish version of the ORTO-15 are not adequate
[12]	adults (sample 1: 404; sample 2: 121)	psychometric validation study	to validate a diagnostic tool for orthorexia nervosa, the ORTO-15 questionnaire	the ORTO-15 questionnaire is a moderately effective tool for identifying orthorexia nervosa in non-clinical populations
[13]	N/A (no empirical sample)	narrative review	to review the current literature on orthorexia nervosa and evaluate its position within the eating disorder spectrum	orthorexia is a potential emerging eating disorder; there’s lack of consensus on diagnostic criteria and classification
[14]	non-clinical sample of adults (313)	prospective longitudinal observational study	to investigate whether ON symptoms predict changes in eating disorder psychopathology and general mental health	ON symptoms predicted increases in eating disorder psychopathology and depressive symptoms over time
[15]	young Italian adults (422)	psychometric validation study	to adapt the Italian version of the Düsseldorf OrthorexiaScale (I-DOS) and to test its psychometric properties	the I-DOS showed good reliability and validity
[16]	competitive athletes from nineteen Olympic sports (273)	cross-sectional observational study	to evaluate the traits of ON and its relation to body composition and anthropometric indices among elite athletes	BMI and body composition are related to ON tendencies in male athletes, whereas weekly training time is related in female athletes
[17]	professional dancers (96)	cross-sectional observational study	to determine the prevalence of ON among professional dancers in Greece, and its relationship with nutrition, BMI, body image flexibility, and parental bonding	disordered eating attitudes and body shape concerns are prevalent among professional dancers
[18]	N/A (no empirical sample)	systematic mini-review	to review studies assessing the prevalence of orthorexia risk in athletes using the ORTO-15 questionnaire	variable prevalence rates of orthorexia risk among athletes; presence of orthorexic tendencies in athletic population
[19]	N/A (no empirical sample)	narrative review	to review existing literature on disinformation about diet and nutrition on social media	dietary misinformation is widespread on social media and can contribute to unhealthy eating behaviors, confusion and distorted nutritional beliefs
[20]	German-speaking sample of young men and women (647)	cross-sectional observational study	to test a social-media based model in the context of orthorexia nervosa	social media users’ involvement with health and fitness accounts is associated with higher orthorexic eating tendencies
[21]	healthy eating websites (HE, 43), pro-eating disorders websites (pro-ED, 24)	qualitative content analysis study	to compare how language and discourse on HE vs. pro-ED websites construct meanings, power relation, and influence beliefs about eating and body practices	both ED and pro-ED websites use similar discursive strategies; pro-ED sites emphasise punishment and devotion, HE could reinforce restrictive and potentially harmful norms
[22]	Chinese university students aged 18 to 25 (2418)	mixed-methods cross-sectional observational study	to examine whether fitness influencers act as positive role models or promote unrealistic/idealized body standards	exposure to fitness influencers is associated with both motivational and negative impacts
[23]	TikTok fitspiration videos (200)	quantitative content analysis study	to examine the content of fitspiration videos on TikTok	videos promoted idealized body types and could reinforce unrealistic body standards
[24]	social media users (680)	cross-sectional quantitative study	to investigate links between Instagram and ON symptoms	higher Instagram use is associated with a greater tendency towards ON
[25]	Instagram post tagged with # orthorexia (3027), Instagram users (185), women having/having had ON (9)	cross-sectional quality study	to study the relationship between ON and Instagram	conversations around # orthorexia on Instagram are associated with the formation of supporting communities aiding recovery
[26]	emerging adults (1446)	cross-sectional observational study	to examine whether use of dietary-focused and physical activity-focused WRSM apps are associated with weight-control and muscle-building behaviors and disordered behaviors	emerging adults who use physical activity- and dietary-focused WRSM apps are more likely to engage in disordered weight-control and muscle-building behaviors
[27]	bodybuilding athletes (107)	quantitative cross-sectional observational study	to investigate behaviors and determinants associated with bodybuilding practices, including the use of dietary supplements and hormones	supplement use is highly prevalent; behavioral and psychological factors are associated with the use of supplements and hormones

**#** = hashtag.

## Data Availability

No new data were created.

## Abbreviations

The following abbreviations are used in this manuscript:

ON	Orthorexia Nervosa
EDs	Eating Disorders
AN	Anorexia Nervosa
BN	Bulimia Nervosa
BED	Binge Eating Disorder
WRSM	Weight-Related Self-Monitoring
DIY	Do It Yourself
DOS	Düsseldorf Orthorexia Scale
